# Including oral health training in a health system strengthening program in Rwanda

**DOI:** 10.3402/gha.v6i0.20109

**Published:** 2013-03-08

**Authors:** Brittany Seymour, Ibra Muhumuza, Chris Mumena, Moses Isyagi, Jane Barrow, Valli Meeks

**Affiliations:** 1 Office of Global and Community Health, Harvard School of Dental Medicine, Boston, MA; 2 Department of Oral Health Policy and Epidemiology, Harvard School of Dental Medicine, Boston, MA; 3 Department of Dentistry, Kigali Health Institute, Kigali, Rwanda; 4 Oncology and Diagnostic Sciences, University of Maryland School of Dentistry, Baltimore, MD

**Keywords:** workforce training, oral health, interdisciplinary, health system strengthening, Rwanda

## Abstract

**Objective:**

Rwanda's Ministry of Health, with the Clinton Health Access Initiative, implemented the Human Resources for Health (HRH) Program. The purpose of the program is to train and retain high-quality health care professionals to improve and sustain health in Rwanda.

**Design:**

In May 2011, an oral health team from Rwanda and the United States proposed that oral health be included in the HRH Program, due to its important links to health, in a recommendation to the Rwandan Ministry of Health. The proposal outlined a diagonal approach to curriculum design that supports the principles of global health through interconnected training for both treatment and collaborative prevention, rather than discipline-based fragmented training focused on isolated risk factors. It combined ‘vertical’ direct patient care training with ‘horizontal’ interdisciplinary training to address common underlying risk factors and associations for disease through primary care, program retention, and sustainability.

**Results:**

The proposal was accepted by the Ministry of Health and was approved for funding by the US Government and The Global Fund. Rwanda's first Bachelor of Dental Surgery program, which is in the planning phase, is being developed.

**Conclusions:**

Competencies, the training curriculum, insurance and payment schemes, licensure, and other challenges are currently being addressed. With the Ministry of Health supporting the dental HRH efforts and fully appreciating the importance of oral health, all are hopeful that these developments will ultimately lead to more robust oral health data collection, a well-trained and well-retained dental profession, and vastly improved oral health and overall health for the people of Rwanda in the decades to come.

While the concept of global health is yet to be universally defined, a number of themes and principles defining global health are widely accepted in the literature. The global health concept seeks an understanding of the global burden of disease and promotes coordinated responses needed through multisectoral and interdisciplinary approaches to sustainable population health. With an appreciation for the role that culture and ethical conduct play, global health demands a broad understanding of linked risks, social determinants, and their impacts on health. It requires integrated prevention-oriented programming and partnership from individual, community, and national levels, with an emphasis on vulnerable populations (1–20). The last decade of milestone global oral health reports and publications demonstrate that concepts important to oral health have evolved to align with those of global health.

Landmark global recognition of oral health as an integral part of overall health was reported in the World Oral Health Report in 2003 (21), with a special bulletin in 2005 devoted to oral health. These documents noted that oral diseases are the most common chronic diseases worldwide, negatively impacting nutrition and growth in children and general well-being of populations (21–24). These reports emphasized the links between oral diseases and systemic diseases, and urged interprofessional prevention with a focus on common risk factors, such as poor diet, stress, lack of clean water, poor sanitation, tobacco, and alcohol abuse (21–25).

Substantial evidence exists linking tobacco use to poor oral health outcomes that negatively impact quality of life at the global level, and as a direct result, oral cancer is the third leading type of cancer in certain countries and the eighth overall (25, 26). Diet plays a significant role, and concern for increased caries rates rises with global adoption of diets high in refined sugars and low in nutritional value. In addition, early signs of malnutrition, such as micronutrient deficiencies, can be seen in the oral cavity (27). As with malnutrition, oral lesions are often early clinical manifestations of the progression of HIV to AIDS, appearing in 80% of those with AIDS (28). The oral manifestations of HIV/AIDS decrease one's ability for adequate nutritional intake, and poor nutrition compounds impaired immune status (27, 28). Global trends, such as an aging population, rising incidence of chronic diseases with oral health associations, greater uptake of tobacco products and sugary foods in low-resource countries, and increased use of medications that cause xerostomia, are predicted to have increased the global burden of oral diseases (24, 29). These examples, while not exhaustive, clearly demonstrate that oral health shares major concerns with other challenges in global health.

Perhaps one of the most significant and historic events for oral health was the United Nations Summit on Non-Communicable Diseases (NCDs) side session devoted to oral health. For the first time, oral diseases were formally recognized by a UN Resolution as a public health problem, noting that ‘renal, oral, and eye diseases pose a major health burden for many countries and that these diseases share common risk factors and can benefit from common responses to non-communicable diseases (30).’ This event highlighted one of the most important principles of global health: that all aspects of health are truly interconnected and require interconnected responses in action. These efforts of the past decade have led to a greater acknowledgement of oral health's importance and awareness of the continued burden of oral diseases. In turn, they have led to a proposal for integrated solutions at a global level.

## Oral health in Rwanda

**Fig.1:**
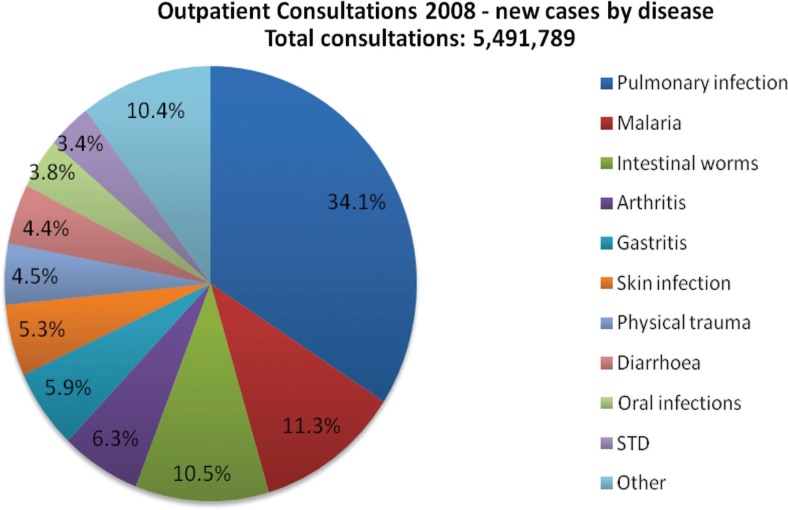
Rwanda's 2008 DHS data for total outpatient consultations in Rwanda's public health centers and hospitals. Source: Rwanda MOH/HMIS Annual Report 2008.

While the exact burden of oral disease in Rwanda is currently unknown, recent Demographic Health Surveys administered by the Ministry of Health and supported by USAID illuminated that oral diseases are a major problem. Nearly 4% of all outpatient consultations in Rwanda's community health centers and hospitals nationwide were related to patient chief complaints regarding oral infections, the mouth, or a tooth problem (Fig. 1). Data for children under the age of five at the district level hospitals identified tooth and mouth problems as 6% of the morbidity of this population. The surveys revealed that in 2011, the leading cause of morbidity in district hospitals were oral diseases, totaling 15% of all cases (Table 1).

**Table 1:** Top 10 causes of morbidity in Rwanda district hospitals in 2011

Diseases	Children < 5 years	Patients ≥5 years	Total cases	Percentage of all cases (%)
Teeth and gum diseases	3,642	79,904	83,546	15
Allergic conjunctivitis	5,769	34,357	40,126	7
Eye diseases	2,066	26,185	28,251	5
Gastro-intestinal disorders	0	20,230	20,230	4
ARIs	5,716	11,293	17,009	3
Skin diseases	2,345	13,864	16,209	3
Gynecological and obstetric diseases	80	15,704	15,784	3
Physical trauma	1,327	14,194	15,521	3
Urinary tract diseases	759	14,633	15,392	3
Epilepsy	1,033	13,083	14,116	3
Other diagnoses	45,940	232,786	278,726	51
Total	68,677	476,233	544,910	100

Source: National HMIS Database, 2011.

Despite this profound oral disease morbidity and treatment need in Rwanda, a severe shortage of adequately trained oral health personnel exists. There are approximately 92 public oral health care providers, which include dentists and dental therapists, serving a country of about 11 million (31). There are only 13 dentists of Rwandan origin who primarily work in urban, public, or private settings, leaving only approximately 10 expatriate dentists, perhaps less, to staff the training institutes and teaching and referral hospitals. Because Rwanda does not have a training program for dentists, all dentists practicing in-country obtained their degrees outside of Rwanda, increasing the risk for what is often referred to as ‘brain drain’ or the inadequate long-term retention of trained dentists in Rwanda.

Because the number of dentists is so limited, most oral care administered in Rwanda is provided by dental therapists. Presently, the Kigali Health Institute (KHI) offers a Bachelor of Dental Therapy (BDT) program wherefrom an annual class of about 27 therapists graduates every year. Currently, dental therapists in Rwanda are trained with a limited scope to provide basic, non-invasive restorative and periodontal procedures, and simple tooth extractions. The 78 dental therapists working in Rwanda practice in government-sponsored facilities. Clearly, with such a limited number of oral health care providers, millions of Rwandans are without adequate care, creating a major obstacle for improved health and economic development.

## Rwanda human resources for health program

In May 2011, Rwanda's Ministry of Health, in partnership with the Clinton Health Access Initiative, invited faculty from 19 United States health professional schools and approximately 60 health sciences faculty from the KHI to Kigali, Rwanda, to announce a major new global health initiative planned for the country. Dr. Agnes Binagwaho, Rwanda's Minister of Health, revealed plans for the Human Resources for Health (HRH) Program, a pioneering initiative that ‘encompasses a partnership with American institutions through to 2019 to ensure that Rwanda's health sciences education system produces the quality and quantity of health professionals and medical faculty necessary to serve the country's population of 11 million and to guarantee a sufficient supply in the future (32).’ In other words, the program is designed specifically to address the greatest challenges to high quality health care in Rwanda. Funding is provided by the United States Government and The Global Fund to Fight AIDS, Tuberculosis, and Malaria.

These challenges include a critical shortage of providers and inadequate health care training and educational programs. US and Rwandan faculty will partner and teach clinical health sciences over a period of 7 years. The ultimate goal is to improve the quality of health care education and increase the number of trained health providers in Rwanda (32). Even though oral health faculty were included in the initial invitation to learn about the program, it was announced during preliminary meetings that dentistry would not be included in the starting phase of the HRH Program.

The oral health team present in Kigali, consisting of three dental faculty members from KHI, one from the University of Maryland School of Dentistry, and two from the Harvard School of Dental Medicine, identified a major gap in Rwanda's current health education system. Presently, Rwanda does not have a formal dental school to train dentists who can adequately meet the oral health needs of the country. The team identified this gap as an opportunity to inform the Ministry of Health and HRH faculty consortium of the importance of oral health and the necessity to include dentistry in the new global health initiative for Rwanda by developing a program to train dentists.

The BDT program alone clearly will not suffice to address the shortages described above because of the therapists’ limited scope of practice and their small numbers. To respond to the disparities in access to oral health care, the oral health team felt that it is critical that Rwanda first establish a formal dental surgery program in Rwanda, producing graduate dentists with a Bachelor of Dental Surgery (BDS) (33).

In response to the Government of Rwanda's comprehensive and innovative HRH Program, the oral health team proposed the induction of a model, first class dental educational and training program geared toward graduating first-rate dental health professionals. Under the HRH Scale-up Plan, the Government of Rwanda seeks to create a dramatic shift in the quantity and skill level of trained health professionals. The oral health team deemed that the establishment of the first dental surgery program in Rwanda could help achieve HRH goals by including dentists who can complement dental therapies through team-based practice in Rwanda's primary health care system. Over time, this approach can ultimately fill the oral health gap and provide a focused investment in the Rwandan people (33).

The oral health team outlined a ‘diagonal’ curriculum concept for the proposed new dental surgery program. The diagonal concept blends horizontal interdisciplinary training opportunities with vertical targeting and discipline-specific training. This diagonal approach to curriculum design is similar to a diagonal strategy for health programming and intervention, combining the use of vertical programs targeted to specific diseases and horizontal programs applying to general, broader health systems strengthening, and common risks and associations for disease (34). The goal of this design is to break down isolated educational silos and fragmented professional training and to incorporate interprofessional educational opportunities (20).

The oral health team felt that this diagonal approach to including oral health training in the HRH Program would maximize resources while also aligning with important global health themes. The proposed concept supports the principles of global health through interconnected training for both treatment and collaborative prevention by addressing common determinants of disease, rather than discipline-based fragmented training focused only on isolated risk factors. The concept recommended ‘vertical’, direct, and discipline-specific patient care training, and ‘horizontal’ interdisciplinary training to address common underlying risk factors and associations for disease as part of primary health care.

**Fig. 2:**
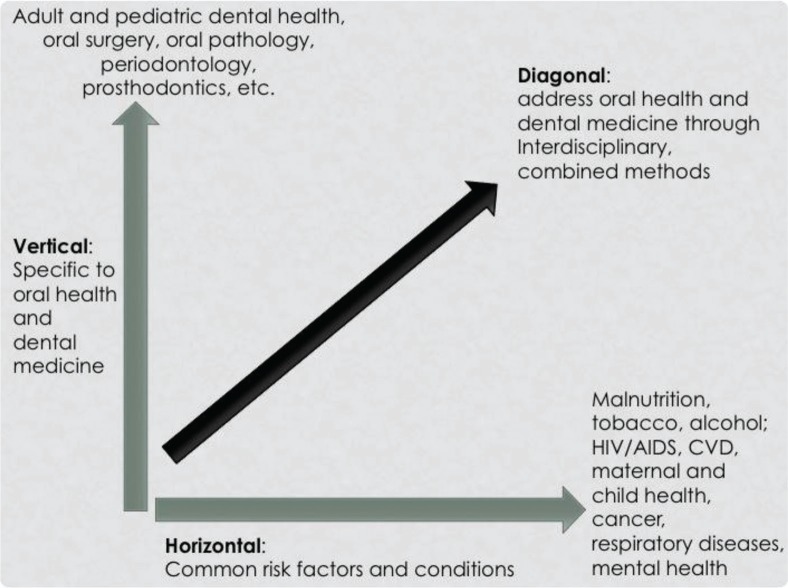
Model of a diagonal curriculum approach for dental medicine and oral health education.

The vertical component of the curriculum concept proposal entailed teaching didactic concepts and clinical skills required specifically for a dental provider. Examples include knowledge and clinical ability to diagnose and treat adult and pediatric dental needs, restorative dentistry, oral surgery, and periodontal therapy. The horizontal component of the curriculum proposal described opportunities for interprofessional education on matters such as common risk factors for a multitude of diseases, the need for sanitation for health, and conditions that affect multiple bodily systems, such as oral conditions, cardiovascular disease, periodontal disease, HIV/AIDS, and diabetes. This combined approach could potentially allow for capacity building and a better understanding of team-based training and practice both within and among multiple health disciplines in the primary health care system. Figure 2 illustrates a sample conceptual framework for this diagonal curriculum concept proposed to the Rwandan Ministry of Health.

## Challenges and progress to date

The Rwandan Ministry of Health accepted the oral health team's proposal to create a curriculum to train the first dentists in Rwanda and has since named oral health as a leading priority. During the first year of implementation of the HRH Program, the oral health team was granted a 1-year planning period, with dentistry to officially become part of the HRH Program in the second year.

With the Rwandan dental faculty leading the effort, intensive planning has begun, and competency and curriculum design for the new BDS program is underway. Detailed competency and curriculum design concepts have undergone an extensive review and revision process by KHI Academic Affairs, Senate, and Board. The University of Rwanda Medical College will house the new BDS program in the newly formed Department of Dentistry; the Medical College Board is also part of the review process. Because the new dental program is a department of the Medical College rather than a separate school, the curriculum in progress is going to allow for co-training of dental and medical students both didactically and clinically, keeping with the diagonal vision of the oral health team. A team of interprofessional faculty from within the HRH faculty consortium has been dedicated to overseeing the *diagonal* approach envisioned. The KHI will continue to provide the BDT program, though the BDT program may be modified as the new BDS program is finalized.

As expected with this type of capacity building program, the oral health team has encountered a number of challenges during the planning year. The HRH Program was designed primarily to enhance existing health education programs. Because Rwanda has never graduated dentists, dentistry has faced a number of unique challenges by creating a new training program altogether. Many questions are still being addressed regarding the scope of practice of the dental therapists versus the dentists, and how reimbursement within the existing national insurance scheme will be structured, for example. How these two types of professionals will complement each other, rather than compete, is currently being deliberated. Legislature mandating the scope of professional practice has not fully been developed, so issues of licensure, legal scope of practice following graduation, and payment structure are carefully being considered. Dental therapists have historically had a level of professional freedom that might face unfamiliar barriers as new legislative mandates are implemented. For example, non-governmental organizations and expatriate dentists have offered advanced skills training to dental therapists in the past that might no longer align with new standards underway for therapists versus dentists.

Other challenges that have arisen include a lack of resources, staff, faculty, and supplies at potential clinical training sites for the dental students. More data on the specific oral health needs of stratified populations in both rural and urban areas must be collected and deployed for systems planning. Current rotation sites for dental therapists must be calibrated for standards of operation that will allow for a productive learning experience for dental students. All of these challenges will continue to be addressed throughout the HRH initiative with input from leading Rwandan dental faculty experts, organized dentistry, and governmental representatives. Dental experts, faculty, and colleagues from neighboring countries have also provided invaluable insight and input into the program design and will consult on implementation.

## Next steps and the way forward

Though much has been accomplished since the original announcement of the HRH Program and convening of the faculty consortium, a great amount of work is yet to be done for the dental component. Final refinement and approval of BDS competencies and the full curriculum must occur at all levels, and integration with other curricula, specifically the medical curriculum, must continue to be refined for the *diagonal* approach to function. Finally, the Ministry of Education's Higher Council of Education and Parliament must ensure that the new curriculum measures up to the quality and standards of higher education in Rwanda.

A clear competency differentiation, licensure process, and payment scheme must be defined and legislated for dental therapists and dentists in Rwanda. Training programs and rotation sites must be modified to complement each other and teach therapists and dentists how to work together, while still providing adequate learning for both programs independently. Team-based interprofessional training opportunities and primary health care clinical rotation sites must continue to be established for all professional students affected by the HRH Program, including dental students.

In the coming months, the application and selection process for the inaugural class of BDS candidates will begin. Though many challenges are still being addressed, and more are expected to arise, the inclusion of oral health and dentistry in the pioneering HRH Program is groundbreaking for Rwanda. With the Ministry of Health supporting the dental HRH efforts and fully appreciating the importance of oral health, all are hopeful that these developments will ultimately lead to more robust oral health data collection, a well-trained and well-retained dental profession, and vastly improved oral health and overall health for the people of Rwanda in the decades to come.
